# Dissociable Patterns in the Control of Emotional Interference in Adults with Attention-Deficit/Hyperactivity Disorder (ADHD) and in Adults with Alcohol Dependence

**DOI:** 10.1371/journal.pone.0107750

**Published:** 2014-09-29

**Authors:** Ivo Marx, John Krause, Christoph Berger, Frank Häßler

**Affiliations:** 1 Department of Psychiatry and Psychotherapy, University Medicine Rostock, Rostock, Germany; 2 Department of Forensic Psychiatry, University Medicine Rostock, Rostock, Germany; 3 Department of Child and Adolescent Psychiatry, Psychotherapy and Neurology, University Medicine Rostock, Rostock, Germany; University of California, San Francisco, United States of America

## Abstract

**Objectives:**

To effectively manage current task demands, attention must be focused on task-relevant information while task-irrelevant information is rejected. However, in everyday life, people must cope with emotions, which may interfere with actual task demands and may challenge functional attention allocation. Control of interfering emotions has been associated with the proper functioning of the dorsolateral prefrontal cortex (DLPFC). As DLPFC dysfunction is evident in subjects with ADHD and in subjects with alcohol dependence, the current study sought to examine the bottom-up effect of emotional distraction on task performance in both disorders.

**Methods:**

Male adults with ADHD (n = 22), male adults with alcohol dependence (n = 16), and healthy controls (n = 30) performed an emotional working memory task (n-back task). In the background of the task, we presented neutral and negative stimuli that varied in emotional saliency.

**Results:**

In both clinical groups, a working memory deficit was evident. Moreover, both clinical groups displayed deficient emotional interference control. The n-back performance of the controls was not affected by the emotional distractors, whereas that of subjects with ADHD deteriorated in the presence of low salient distractors, and that of alcoholics did not deteriorate until high salient distractors were presented. Subsequent to task performance, subjects with ADHD accurately recognized more distractors than did alcoholics and controls. In alcoholics, picture recognition accuracy was negatively associated with n-back performance, suggesting a functional association between the ability to suppress emotional distractors and successful task performance. In subjects with ADHD, performance accuracy was negatively associated with ADHD inattentive symptoms, suggesting that inattention contributes to the performance deficit.

**Conclusions:**

Subjects with ADHD and alcoholics both display an emotional interference control deficit, which is especially pronounced in subjects with ADHD. Beyond dysfunctional attention allocation processes, a more general attention deficit seems to contribute to the more pronounced performance deficit pattern in ADHD.

## Introduction

Attention-Deficit/Hyperactivity Disorder (ADHD) is a childhood-onset syndrome consisting of inattentive, hyperactive, and impulsive characteristics [1]. Approximately 65% of children and adolescents with ADHD show partial remission by the age of 25, whereas 15% continue to meet full DSM-IV criteria [2]. Given a prevalence rate of 2.9% to 4.4%, ADHD is one of the most common psychiatric disorders in adulthood [3], [4]. Compared to children with ADHD, affected adults show fewer hyperactive symptoms, but their inattentive and impulsive symptoms persist [5]. These symptoms cause complications in areas such as work, social life, and relationships [6], [7]. Subjects with ADHD are often susceptible to alcoholism [8]–[10]. It has been shown that the risk of developing an alcohol use disorder is increased by a factor of 1.7 in children with ADHD [11]. Furthermore, adult alcoholics with ADHD have been found to begin to exceed the critical level of alcohol use earlier [8] and show a pattern of longer-lasting abuse [12] compared with non-ADHD alcoholics.

Deficits in emotion regulation (ER) have been reported in children and adolescents [13]–[17] as well as in adults with ADHD (for a review, see [18]). ER deficits seem to be prominent especially in the combined subtype [13]–[14], which suggests an association with hyperactive-impulsive symptoms and therefore with deficient inhibitory control of inadequate emotional expression. Studies that have focused on the expressive aspect of emotional dysregulation in children with ADHD have found an increased expression of negative affect in potentially frustrating situations [14], [15], [17]. Importantly, emotional dysregulation has been negatively associated with social status and peer preference [14], [15], which underlines the relevance of these deficits for everyday life. Likewise, deficits in ER have been reported in alcohol-dependent subjects [19], [20] and have been linked with alcohol dependency severity [21], [22]. Because the severity of alcohol dependency has been related to inhibition deficits [23], a connection between deficient inhibitory capacities and impaired ER is suggested. Thus, ER deficits may be caused by deficient inhibitory control over emotional responses in both ADHD and alcohol dependence.

Barkley [24] suggests that deficits in emotional self-regulation in subjects with ADHD arise from increased emotional impulsivity (i.e., a lowered threshold and increased speed of responding to emotional cues), which itself is the result of a lack of effortful executive control. Executive control thus does not reflect a unitary construct but rather represents an accumulation of cognitive functions that subserve adaptive, goal-directed behavior. For example, executive functions encompass the allocation of attention toward task-relevant information, the inhibition of task-irrelevant information, planning, and behavior monitoring (for reviews, see [25], [26]). In Barkley's model [24], self-regulation, or executive control, is conceptualized as a sequential construct that incorporates the inhibition of emotion-induced inappropriate behavior, self-calming, attention refocusing, and organized goal orienting. Likewise, Nigg and Casey [27] argue that ER deficits in ADHD are the result of a dysregulation of the frontolimbic circuitry that incorporates the amygdala, ventral striatum, and prefrontal top-down modulatory areas. Expanding the network of emotion processing according to the current state of research in healthy subjects, the regions most commonly involved in the generation of emotions involve the amygdala, the ventral striatum, the ventromedial prefrontal cortex, and the insula. However, executive control is exerted mainly by the dorsolateral prefrontal cortex (DLPFC), the anterior cingulate cortex, the ventrolateral prefrontal cortex, and the dorsomedial prefrontal cortex (for a review, see [28]).

Despite the fact that subjects with ADHD and subjects with alcohol dependence seem to suffer from top-down ER deficits, little is known about their prerequisites, the bottom-up effects of dysfunctional emotion processing. In this sense, deficient attentional control of interfering emotional stimuli or a lowered threshold for emotional cues [24] might cause emotional over-reagibility, which, in turn, might be a prerequisite for subsequent deficits in effortful behavioral control. Attentional control is thus defined as the ability to orient attention to task-relevant information while inhibiting task-irrelevant information [29]–[31], a function that has been shown to rely on the DLPFC [32].

One way to examine the attentional control of interfering emotional stimuli in experimental contexts is by using paradigms of emotional interference control. In these paradigms, cognitive tasks are imposed that require a continuous orienting of attention to task-relevant information. While the subjects work on the task, emotionally salient but task-irrelevant distractors are imposed, and an experimenter examines how these distractors interfere with attentional control (i.e., the degree to which performance deteriorates in the distractor's presence when compared with its absence) [33]. Assuming a limited number of attentional resources in the individual, task-irrelevant but emotionally salient distractors interfere with the primary task and cause a “fight for resources”. In this way, the bottom-up effect of emotional distraction and a lack of cognitive control cause an over-allocation of resources to the emotional distractors and an under-allocation of resources to the primary task, which results in impaired task performance. According to this “neuro-competitive” model introduced by Iordan, Dolcos, and Dolcos [34], increased susceptibility to emotional interference is believed to be grounded in an imbalance of activity between the dorsal executive control system (incorporating the DLPFC and the lateral parietal cortex) and the ventral emotion processing system (incorporating the amygdala, the ventrolateral prefrontal cortex, and the medial prefrontal cortex), whereby a hypofunction of the executive control system causes impaired executive control over emotional responses. This model is supported by recent meta-analytic findings [35] that have identified the neuronal structures that are responsible for emotion processing (amygdala, superior temporal gyrus, insula, medial anterior cingulate cortex), cognitive control (supplementary motor area, superior parietal lobule, DLPFC), and the integration of emotion processing and cognitive control processes (subgenual anterior cingulate cortex, precuneus). Furthermore, the authors found the DLPFC and the inferior parietal lobule to be specifically involved in the processing of task-irrelevant emotional distractors, which underlines their unique role in attention allocation processes.

In the current study, we implemented a working memory (“n-back”) task that requires attentional control as exerted by the DLPFC [36]. At the same time, we induced emotional interference by presenting pictures of varying emotional salience while participants performed the working memory task. Given its key role in emotional interference control by attention allocation processes [35], DLPFC functionality should be affected by emotional content; that is, emotionally salient but task-irrelevant stimuli should withdraw attention from the working memory task and should result in poorer task performance in terms of prolonged response times and decreased performance accuracy.

Testing this hypothesis in adults with ADHD, Marx et al. [37] recently found that those with the disorder displayed enhanced distractibility by emotionally salient stimuli in terms of lower performance accuracy when compared with non-affected controls. Specifically, the performance of subjects with ADHD deteriorated in the presence of emotionally low salience distractors, but that of the controls did not deteriorate until emotionally high salience distractors were presented. However, to our knowledge, no comparable research examining emotional interference control in alcoholics exists. Because working memory (WM) deficits have been demonstrated in individuals with ADHD [38], [39] and in individuals with alcoholism [40], suggesting DLPFC dysfunction, and because both disorders show ER deficits, it remains unclear whether the disorders share a common dysfunction in the emotion processing system or whether deficient emotional interference control is specific to ADHD. WM deficits have been shown to be comparably stable in the course of ADHD [40], [41], but these deficits seem to vary as a function of abstinence duration in alcohol-dependent individuals, with medium-sized effects emerging for short-term (up to one month) and intermediate-term (up to one year) abstinence and small effect sizes emerging for long-term abstinence (more than one year) [40]. Thus, WM deficits seem “trait-like” in adults with persisting ADHD and “state-like” in adults with alcohol dependency depending on the duration of abstinence, such that subjects with ADHD might be more susceptible to emotional interferences.

The current study aims to compare adults with ADHD (without comorbid alcohol dependence) and adults with alcohol dependence (without comorbid ADHD) relative to healthy control subjects in their ability to cope with emotional interferences while performing an emotional working memory (“n-back”) task. First, we expect all groups to display performance deterioration as a function of task difficulty and the degree of distractor salience. Second, we expect individuals with ADHD and alcoholic individuals to display WM deficits in comparison to controls. Third, because the two disorders have emotion processing deficits in common, we expect both groups of patients to be more distractible by task-irrelevant emotional stimuli, as indicated by larger performance deterioration under emotionally arousing conditions in comparison to controls. Fourth, because DLPFC dysfunction seems to be more stable over time in ADHD, the emotional interference effect should be more accentuated in the ADHD group compared with the alcoholism group. Fifth, because enhanced emotional distractibility implies a larger amount of attention allocation to emotional distractors, we expect that the ADHD group and alcoholic individuals will memorize the emotional stimuli better than the control group will, with alcoholic subjects outperforming those with ADHD.

## Methods

### Participants

As there is no prior knowledge of gender differences in emotional interference control in subjects with ADHD and in subjects with alcohol dependence, we decided to only include males to control for potential confounding variables associated with gender. The inclusion of males only was driven by sampling considerations. The gender ratio is balanced in adults with ADHD [42], while it is increased by a factor of two to three in alcoholics in favor of males [43]–[45]. Moreover, comorbid disorders hardly differ between alcohol-dependent males and females [44], whereas gender-related differences are present in subjects with ADHD [46], [47]. This is another potentially confounding variable that we aimed to control for.

The study sample consisted of 68 male participants (22 subjects with ADHD, 16 subjects with alcohol dependence, and 30 healthy controls) who were 18 to 45 years old. The participants in the ADHD condition were recruited from the ADHD outpatient service of the Department of Psychiatry and Psychotherapy at the University Medicine Rostock. University hospital employees and students who were recruited via announcements served as controls. All participants with ADHD and 21 controls represent the male subsample of subjects who took part in the previously published study by Marx et al. [37]. Inpatients engaging in detoxification at the Department of Psychiatry and Psychotherapy and inpatients from a local specialist hospital for addictive disorders (Friedrich-Petersen-Klinik) formed the group of subjects with alcohol dependence.

The diagnostic procedure for all of the participants included extensive psychiatric examination using the Structured Clinical Interview for DSM-IV Axis I and II Disorders (SCID) [48] and the Personality Styles and Disorders Inventory (PSSI) [49]. Adult ADHD was assessed using German versions of the Barkley Interview [50], the Wender-Reimherr Interview [51], and the following questionnaires: a short version of the Wender Utah Rating Scale (WURS-k) [52], which was used to quantify ADHD symptoms in childhood; the Conners' Adult ADHD Rating Scales (CAARS-S:L) [53]; a short self-rating questionnaire based on the DSM-IV criteria (ADHS-SB) [51], which was used to assess current ADHD symptoms; and the Barratt Impulsiveness Scale (BIS-10) [54]. The ADHD diagnosis was assigned by a senior clinical psychiatrist and included the following criteria: a WURS-k sum score ≥30 points [55]; an age- and gender-adjusted total ADHD symptom subscale score of ≥1.5 SD above the mean on the CAARS-S:L; substantial impairment in more than one setting; and clinically relevant psychological strain. Alcohol dependence was diagnosed by means of the SCID, and additional information was gathered concerning the year of first alcohol consumption, the amount of alcohol consumed prior to treatment, the number of detoxifications, and the number of other alcohol-related treatments. In addition, the Alcohol Withdrawal Scale (AWS) [56] was used to assess whether alcohol withdrawal symptoms were currently observable in the participants. Participants who scored above 0 were excluded from the study. The intellectual capacity of all subjects was assessed with a short version of the Wechsler Adult Intelligence Scale-Revised (HAWIE-R) [57]. The alcohol-dependent and control subjects underwent the same diagnostic procedure as the subjects with ADHD.

The exclusion criteria for all of the participants included an IQ lower than 80, a neurological or endocrine disorder known to affect brain function, previous head injury, current depressive disorder, lifetime schizophrenia spectrum disorder, and borderline personality disorder. Furthermore, severe cognitive dysfunction (amnestic syndrome, dementia), current intoxication, symptoms of alcohol withdrawal, and severe impairment of liver functioning as measured by the Gamma GT served as exclusion criteria in the alcohol dependency group. Additionally, addiction in the ADHD group and in the control group as well as ADHD in the alcohol dependency group and in the control group served as exclusion criteria.

In the ADHD group, 16 subjects were combined subtype, five were primarily inattentive, and one subject was primarily hyperactive/impulsive. Half of the subjects were drug naïve; the others had taken methylphenidate previously but were free of any medication for a minimum of 72 h prior to testing. Within the ADHD group, one subject suffered from adjustment disorder, and seven subjects had a personality disorder (other than BPD). In the alcohol dependence group, one subject suffered from a panic disorder, one suffered from a THC addiction, two suffered from polytoxicomania, and three had a personality disorder (other than BPD). The three experimental groups did not differ from each other with regard to age, but subjects with alcohol dependence had lower IQ values compared with the ADHD and control subjects. It should be noted that the relatively high IQ values could be due to the use of the short version of the WAIS, which depends on relatively old German norms. Rather than examining a group of only well-educated subjects, educational and occupational levels were mixed in the experimental groups. The subjects with ADHD displayed increased ADHD symptom severity compared with the alcohol-dependent subjects, and the alcohol-dependent subjects displayed increased ADHD symptom severity compared with the controls. The basic demographic and clinical sample characteristics are shown in Table 1.

**Table 1:** Basic Demographic and Clinical Characteristics.

	ADHD		ALC		CON		F(2,65)	Post-Hoc
	M	SD	M	SD	M	SD		
Age	28.23	6.09	32.60	6.43	29.51	7.79	1.88	
IQ	128.14	17.44	113.44	16.92	126.79	13.55	4.83**	ALC<CON, ADHD
CAARS-S:L							F(2,62)	
Inattention	90.05	22.84	55.35	29.59	32.80	29.15	25.06***	CON <ALC <ADHD
Hyperactivity/Impulsivity	76.95	31.77	43.11	32.74	18.23	26.57	22.74***	CON<ALC<ADHD
ADHD Total	88.66	23.74	52.91	30.42	20.33	23.96	41.73***	CON<ALC<ADHD
Drinking units per day before admission			31.56	19.00				
Years since ≥ 3 DSM criteria fulfilled			8.88	5.37				
Number of detoxifications			1.69	2.60				
Number of other alcohol-related treatments			0.81	1.05				

ADHD =  Subjects with ADHD, ALC =  Alcoholics, CON =  Controls. M =  Mean, SD =  Standard Deviation. *p<.05, **p<.01, *** p<.001.

### Experimental Tasks and Measures

Initially, a classical n-back task was applied. In this task, participants are presented with stimuli and are asked to indicate whether the currently presented stimulus is the same as the one presented n steps earlier in the sequence. The task consisted of eight 1-back and eight 2-back blocks, which were presented randomly. Each block consisted of 14 letters (4 targets, 10 distractors), and the subjects were asked to indicate targets by pressing the left mouse button (“yes”) and distractors by pressing the right mouse button (“no”). The respective n-back condition was indicated by the number “1” or “2”, which appeared at the beginning of each block. The entire task was preceded by two practice trials for each n-back condition.

Subsequently, the participants performed the emotional variant of the task [37]: they worked on the n-back task while simultaneously being presented with emotional background picture stimuli. The subjects were explicitly instructed to ignore the emotionally laden pictures and to process the task as quickly and as accurately as possible. The task consisted of nine 1-back and nine 2-back blocks, where each n-back condition consisted of 3 blocks with 14 neutral, low emotionally salient, or high emotionally salient pictures. Each block contained 14 randomly presented stimuli (4 targets, 10 distractors), and both the pictures within the blocks and the blocks themselves were presented in random order. Each picture was presented for a duration of 3,000 ms. After 1,250 ms had elapsed, the n-back letter was presented in the middle of the screen for a duration of 500 ms. In total, 252 affective pictures were presented, with ratings ranging from “1” (negative valence; low arousal) to “9” (positive valence; high arousal): 84 neutral (valence: M = 5.25, SD = 0.25; arousal: M = 2.39, SD = 0.84), 84 low salience negative (valence: M = 2.67, SD = 0.49; arousal: M = 5.14, SD = 0.49), and 84 high salience negative pictures (valence: M = 2.15, SD = 0.49; arousal: M = 6.39, SD = 0.45). All of the negative and 32 of the neutral pictures were taken from the International Affective Picture System [58], and 52 of the neutral stimuli were collected and evaluated in the department's own research laboratory. The stimuli will be provided upon request to the corresponding author.

After completing the emotional n-back task, all of the subjects performed a surprise recognition memory task in which all 256 pictures that had been used in the first task and 54 new pictures (18 per salience category) were presented in randomized order. The participants were asked to indicate on a four-point scale whether the picture had been presented before (1 =  no, certain 2 =  no, uncertain, 3 =  yes, uncertain, 4 =  yes, certain).

### Dependent Variables

For both versions of the n-back task, the median of the reaction time for correctly identified targets (RT) and the individual discrimination indices for performance accuracy (d′) were used as dependent variables, with the d′ calculated based on the signal detection theory as follows: d′ = z (hits/number of targets) – z (false alarms/number of distractors). The resulting value range was -4.66≤x≤+4.66, with more negative values indicating poorer performance. For the recognition task, the ratings were binarized (known and unknown) prior to computation of the d′.

### Covariates of Interest

*The Questionnaire of Current Motivation* (QCM) [59] is an 18-item questionnaire that was designed to assess the current motivational aspects of task completion, including the dimensions of task interest, perceived challenge, mastery confidence, and incompetence fear, according to the cognitive-motivational process model [60]. Subjects are asked to evaluate the items on a seven-point Likert rating scale (from 1 =  disagree to 7 =  agree), with higher scores reflecting higher task-related motivation. Differences in education history (subjects with ADHD more often attend special schools, repeat grades, receive special educational services and are less likely to graduate from school) [61], [62], self-esteem, and control beliefs (subjects with ADHD have a more external locus of control and suffer from lower self-esteem) [63]–[65] may differentially affect task-related motivation in experimental groups; therefore, the QCM was considered a possible covariate of interest in the present study to rule out systematic motivational group differences that might have existed prior to the experimental manipulation of reward options in the present study and that might independently impact the dependent variables. The prognostic value of the QCM was demonstrated by showing that mastery confidence and incompetence fear predicted learning performance in a complex, computer-simulated system [60]. Moreover, the task properties (self-regulated vs. question guided) and characteristics of the subject (“slow learners” vs. “fast learners”) were demonstrated to mediate the association of task interest and challenge with learning success [59]. In this study, we adopted the following QCM items: (1) “I enjoy doing this kind of task,” (2) “The task seems very interesting to me,” (3) “I'm very curious about how well I will perform the task,” and (4) “I'm a little bit scared that I could make a fool of myself”. The subjects answered these four items subsequent to the practice trial. The individual mean score, with item four inverted, served as an indicator of the potential motivational group differences in task engagement.

### Procedure

The examination took place at the research laboratory of the Department of Psychiatry and Psychotherapy, University Medicine Rostock. After the study purposes were explained to the subjects non-specifically, the subjects were seated in the quiet and dimmed experiment room and worked on the experimental tasks. Both of the n-back tasks were projected onto a white screen using a Sanyo 285 LCD Z2 projector. The subjects' distance to the screen was approximately 138 inches, and the screen had a width of 51 inches. The visual angle was 21 degrees. The experimental paradigm was presented using presentation software (Neurobehavioral Systems Inc., Berkeley, CA, USA), which was installed on a desktop PC (Compaq HP dc 5700; CPU: Pentium 4, 3 GHz; 1 GB RAM). The total duration of the test was approximately 60 minutes.

The study was conducted in accordance with the latest version of the Declaration of Helsinki. The ethics committee of the Faculty of Medicine of the University of Rostock approved this study (registration number: A 2012–0020). The subjects were provided with information about the task to be performed and the study procedure, and written informed consent was obtained from all of the participants. All of the subjects were able to consent (i.e., they were able to understand, reproduce, and draw a rational conclusion based on the information provided in the written subject information). Subjects who did not have the capacity or ability to consent were excluded according to the exclusion criteria.

### Statistical Analyses

The data were analyzed using SPSS version 17 (SPSS Inc., Chicago, IL, USA). Tests of the group differences in sociodemographic and clinical data and task-related motivation were performed using univariate analyses of variance (ANOVAs). The group differences in the dependent variables (RT, d′) were analyzed using repeated-measures ANCOVAs with the diagnostic group (subjects with ADHD; alcohol-dependent subjects; controls) as the between-subjects factor, the n-back level (1-back; 2-back) and emotional salience (neutral; low emotionally salient; high emotionally salient) as the within-subject factors, and IQ as the covariate. In cases of significant main or interaction effects, post-hoc group comparisons were conducted. Multiple comparisons were corrected using the Bonferroni procedure. Prior to the analyses, the raw data were z-transformed to examine extreme outliers (z>3.0). Two extreme outliers were detected and were replaced by the respective group means. The significance level for all of the tests was p≤0.05. The partial eta-squared (*η*_p_^2^) is reported as a measure of the effect size.

## Results

Because psychostimulants have been demonstrated to improve working memory performance [66] by improving prefrontal cortex activity [67], [68], we compared the drug-naïve ADHD subjects with those who had discontinued their medication in relation to the dependent variables in the classical n-back task prior to all subsequent analyses. No subgroup differences were observed for RT or d′ (all *p*s>0.05). Importantly, the ADHD subgroups did not differ from each other with regard to age, *t*(20)  = −0.07, *p* = .95, *ns*, intellectual capacity, *t*(20)  = −0.20, *p* = .84, *ns*, or task-related motivation, *t*(20)  = 1.00, *p* = .33, *ns* and were thus analyzed as a group.

### Task-Related Motivation

The experimental groups did not differ from each other in terms of their initial task-related motivation, *F*(2,65)  = 1.97, *p* = .15, *ns*. The three experimental groups displayed an intermediate motivational level (subjects with ADHD: *M* = 4.32, *SD* = 1.17; alcoholics: *M* = 4.45, *SD* = 1.35; controls: *M* = 4.94, *SD* = 1.11).

### Classical N-back Task

**Figure 1:**
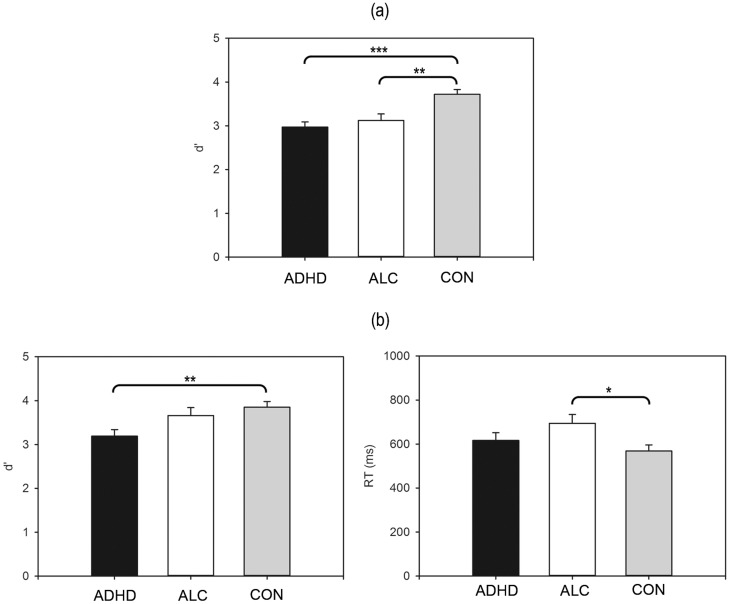
Between-Subjects Effect for the Classical N-back Task (a); Between-Subjects Effects for the Emotional N-back Task (b). ADHD = ADHD group, ALC =  alcoholics, CON =  control group. d′ =  discriminability score, RT =  reaction time in milliseconds. *p<.05, **p<.01, ***p<.001.

No group differences for RT emerged (all *p*s>0.05), but a between-subjects effect of working memory load was found for d′, *F*(2,63)  = 12.17, *p*<.001, *η*_p_^2^ = .28, and post-hoc comparisons indicated that subjects with ADHD and alcohol-dependent subjects were both impaired relative to controls (CON vs. ADHD: *p*<.001; CON vs. ALC: *p* = .007; ADHD vs. ALC: *p* = 1.00) (see Figure 1). No further significant effects emerged (all ps>0.05). The mean reaction times and accuracy scores for the classical n-back task are presented in Table 2.

**Table 2.** Descriptive Statistics for the Classical N-back Task Performance.

	ADHD		ALC		CON		Group	N-back	N-back x Group
	M	SD	M	SD	M	SD	F(2,63)	F(1,63)	F(2,63)
Reaction Time (ms)							1.70	0.81	0.88
1-back	591.83	140.22	637.59	202.54	558.05	153.82			
2-back	729.51	237.42	762.55	215.01	639.87	216.98			

							F(2,63)	F(1,63)	F(2,63)
Performance Accuracy (d′)							12.17***	3.38^(*)^	0.57
1-back	3.26	0.44	3.09	0.88	3.92	0.54			
2-back	2.81	0.87	2.81	0.95	3.62	0.82			

ADHD =  Subjects with ADHD, ALC =  Alcoholics, CON =  Controls. Group  =  between-subjects effect of experimental group membership, N-back  =  within-subjects effect of task difficulty, N-back x Group  =  within-between-subjects interaction effect. M =  Mean, SD =  Standard Deviation. ^(^*^)^p<.10, *p<.05, **p<.01, *** p<.001.

### Emotional N-back Task

**Figure 2:**
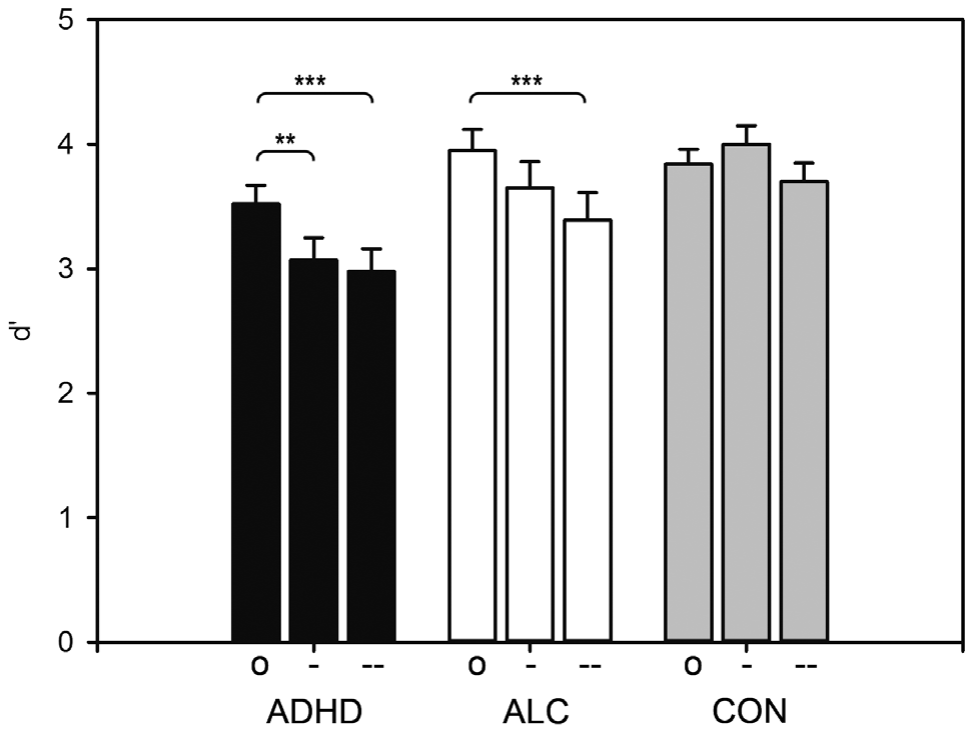
Experimental Group x Distractor Salience Level Interaction Effect for the Emotional N-back Task. ADHD = ADHD group, ALC =  alcoholics, CON =  control group. o =  neutral distractors, -  =  low emotionally salient distractors, - -  =  high emotionally salient distractors. d′ =  discriminability score. *p<.05, **p<.01, ***p<.001.

Working memory load affected d′, *F*(1.61)  = 5.97, *p* = .02, *η*_p_^2^ = .09, with all subjects responding less accurately in the 2-back condition compared with the 1-back condition. Likewise, the emotional salience of the background picture stimuli affected d′, *F*(2,122)  = 3.26, *p* = .04, *η*_p_^2^ = .05, in all subjects. The post-hoc analyses revealed that performance accuracy decreased gradually when the salience of the background pictures increased (neutral vs. low salience pictures: *p* = .04; neutral vs. high salience pictures: *p*<.001; low salience vs. high salience pictures: *p* = .04). Group differences emerged both for RT, *F*(2,60)  = 3.25, *p* = .05, *η*_p_^2^ = .10, and for d′, *F*(2,61)  = 5.84, *p* = .005, *η*_p_^2^ = .16. Alcohol-dependent subjects displayed prolonged RTs relative to controls (CON vs. ADHD: *p* = .85, *ns*; CON vs. ALC: *p* = .04; ADHD vs. ALC: *p* = .47, *ns*), and subjects with ADHD displayed decreased d′ compared with controls (CON vs. ADHD: *p* = .004; CON vs. ALC: *p* = 1.00, *ns*; ADHD vs. ALC: *p* = .16, *ns*) (see Figure 1). Furthermore, an emotion x group interaction effect emerged both for RT, *F*(4,120)  = 3.75, *p* = .007, *η*_p_^2^ = .11, and for d′, *F*(4,122)  = 3.56, *p* = .009, *η*_p_^2^ = .10. RTs differed between the groups when high salience pictures were presented, *F*(2,65)  = 3.21, *p* = .05, *η*_p_^2^ = .10, but the subsequently conducted post-hoc tests yielded insignificant results (all *p*s>.05). When emotional distractor salience increased, performance accuracy deteriorated in subjects with ADHD, *F*(2,38)  = 11.08, *p*<.001, *η*_p_^2^ = .37, and in alcohol-dependent subjects, *F*(2,30)  = 6.05, *p* = .006, *η*_p_^2^ = .29, but not in controls, *F*(2,58)  = 2.79, *p* = .07, *ns*. The performance of subjects with ADHD deteriorated in the presence of low salience pictures (neutral vs. low salience pictures: *p* = .009; neutral vs. high salience pictures: *p*<.001; low salience vs. high salience pictures: *p* = 1.00, *ns*), whereas that of alcohol-dependent subjects did not deteriorate until high salience pictures were presented (neutral vs. low salience pictures: *p* = .84, *ns*; neutral vs. high salience pictures: *p* = .001; low salience vs. high salience pictures: *p* = .20, *ns*) (see Figure 2). No further effects were significant (all *p*s>0.05). The mean reaction times and accuracy scores for the emotional n-back task can be derived from Table 3.

**Table 3:** Descriptive Statistics for the Emotional N-back Task Performance.

	ADHD		ALC		CON		Group	N-back	N-back x Group	Emotion	Emotion x Group
	M	SD	M	SD	M	SD	F(2,60)	F(1,60)	F(2,60)	F(2,120)	F(4,120)
Reaction Time (ms)							3.25*	0.43	0.44	0.55	3.75**
1-back											
neu	568.97	133.42	633.38	158.32	535.26	153.06					
low	549.50	149.97	674.90	158.63	534.39	147.44					
high	632.89	168.32	692.43	134.38	581.55	159.67					
2-back											
neu	676.51	214.79	725.01	167.03	585.75	190.45					
low	580.01	157.57	672.03	188.44	584.76	171.96					
high	711.76	236.21	719.69	150.51	599.79	185.79					

							F(2,61)	F(1,61)	F(2,61)	F(2,122)	F(4,122)
Performance Accuracy (d′)							5.84**	5.97*	2.87^(*)^	3.26*	3.56**
1-back											
neu	3.84	0.57	3.92	0.75	4.06	0.66					
low	3.37	1.01	3.60	0.76	4.10	0.53					
high	3.36	0.98	3.29	1.04	3.83	0.84					
2-back											
neu	3.34	1.34	3.62	0.99	3.72	0.69					
low	2.85	1.51	3.53	0.72	3.94	0.74					
high	2.76	1.30	3.08	0.85	3.68	0.89					

ADHD =  Subjects with ADHD, ALC =  Alcoholics, CON =  Controls. Group  =  between-subjects effect of experimental group membership, N-back  =  within-subjects effect of task difficulty, N-back x Group  =  within-between-subjects interaction effect, Emotion  =  within-subjects effect of the emotional salience of the background picture stimuli, Emotion x Group  =  within-between-subjects interaction effect. M =  Mean, SD =  Standard Deviation. neu  =  neutral pictures, low  =  low emotional salience, high  =  high emotional salience. ^(^*^)^p<.10, *p<.05, **p<.01, *** p<.001.

### Surprise Recognition Memory Task

**Figure 3:**
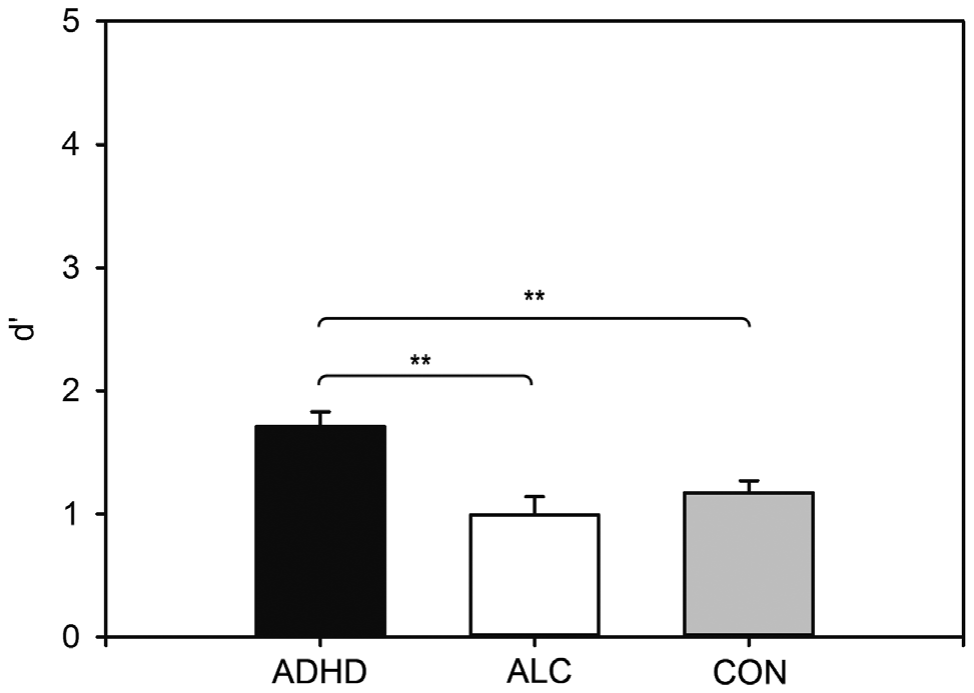
Between-Subjects Effect for the Surprise Memory Recognition Task. ADHD = ADHD group, ALC =  alcoholics, CON =  control group. d′ =  discriminability score. *p<.05, **p<.01, ***p<.001.

The three experimental groups differed significantly in their picture recognition performance, *F*(2,56)  = 8.73, *p*<.001, *η*_p_^2^ = .24, with subjects with ADHD outperforming both alcohol-dependent subjects and controls (CON vs. ADHD: *p* = .003; CON vs. ALC: *p* = 1.00, *ns*; ADHD vs. ALC: *p* = .002) (see Figure 3). No further effects were significant (all *p*s>0.05). The accuracy scores for the surprise recognition memory task are provided in Table 4. All between-subjects effects and between-within interaction effects are displayed in Figure 1.

**Table 4:** Descriptive Statistics for the Surprise Recognition Memory Task.

	ADHD		ALC		CON		Group	N-back	N-back x Group	Emotion	Emotion x Group
	M	SD	M	SD	M	SD	F(2,56)	F(1,56)	F(2,56)	F(2,112)	F(4,112)
Performance Accuracy (d′)							8.73***	0.06	2.80^(*)^	1.05	0.93
1-back											
neu	1.69	0.69	0.98	0.52	1.14	0.60					
low	2.09	0.84	1.05	0.73	1.57	0.48					
high	2.11	0.81	1.31	0.69	1.61	0.69					
2-back											
neu	1.16	0.69	0.68	0.59	0.62	0.56					
low	1.65	0.84	0.84	0.79	1.02	0.52					
high	1.61	0.65	1.00	0.81	1.07	0.51					

ADHD =  Subjects with ADHD, ALC =  Alcoholics, CON =  Controls. Group  =  between-subjects effect of experimental group membership, N-back  =  within-subjects effect of task difficulty, N-back x Group  =  within-between-subjects interaction effect, Emotion  =  within-subjects effect of emotional salience of the background picture stimuli, Emotion x Group  =  within-between-subjects interaction effect. M =  Mean, SD =  Standard Deviation. neu  =  neutral pictures, low  =  low emotional salience, high  =  high emotional salience. ^(^*^)^p<.10, *p<.05, **p<.01, *** p<.001.

### Association between N-Back Task Performance and Picture Recognition Accuracy

Overall performance accuracy as measured by the average d′ across all experimental conditions was negatively associated with subsequent picture recognition in alcohol-dependent subjects (*r* = −0.53, *p* = .05) but not in subjects with ADHD (*r* = −0.15, *p* = .55, *ns*) or controls (*r* = 0.04, *p* = .85, *ns*).

### Association between N-Back Task Performance and Inattentive and Hyperactive/Impulsive Symptoms

The correlation analyses between the CAARS-S:L DSM-IV inattention and hyperactivity/impulsivity subscale sum scores and overall performance accuracy revealed that in subjects with ADHD (*r* = −0.50, *p* = .03) and in controls (*r* = −0.37, *p* = .05), but not in alcohol-dependent subjects (*r* = 0.09, *p* = .75, *ns*), d′ was negatively correlated with inattention, whereas no significant correlations emerged for hyperactivity/impulsivity (all *p*s>0.05).

## Discussion

The current study sought to examine the pattern of emotional interference control in adult males with ADHD and in adult males with alcohol dependence. Given the role of the DLPFC in coping with emotional distractors and based on the knowledge of common but differentially pronounced DLPFC dysfunction in subjects with ADHD and in subjects with alcohol dependence, we expected both clinical groups to display WM deficits and emotional interference control deficits when compared with controls, with the emotional interference control deficit being more accentuated in those with ADHD.

As expected, WM deficits were observed in both clinical groups, with a dissociation of the quality of this deficit emerging in the emotional version compared with the classical version of the task. Whereas both clinical groups displayed decreased performance accuracy compared with the controls in the classical task, subjects with ADHD displayed decreased performance accuracy but alcohol-dependent subjects displayed increased response times in the emotional version of the task. This effect might be interpreted as indicating that alcohol-dependent subjects, by taking more time to make their decisions, were able to ensure performance quality that paralleled that of the control group, suggesting a less pronounced WM deficit in alcohol-dependent subjects.

Moreover, a deficit in coping with emotional interferences was found in both clinical groups. Whereas the controls displayed stable performance accuracy across all salience levels (i.e., they were not at all susceptible to the emotional content of the implemented pictorial distractors), the performance of subjects with ADHD deteriorated in the presence of low salience pictures, whereas that of alcohol-dependent subjects did not deteriorate until high salience pictures were presented. Thus, the emotional interference control deficit was more pronounced in subjects with ADHD. This interpretation is underlined by three facts: (1) Subjects with ADHD accurately recognized more distractor pictures from the emotional n-back task subsequent to task performance, indicating larger attention allocation to the emotional distractors or, in other words, impaired interference control relative to alcoholics and controls. (2) Picture recognition accuracy was negatively associated with WM performance in the alcoholic group but not in the ADHD and control groups, suggesting an association between the ability to suppress emotional distractors and success in n-back task performance solely in alcohol-dependent subjects. This association might be lacking in the controls because they fully concentrated on the primary task, as suggested by their lack of susceptibility to the emotional distractors, whereas subjects with ADHD might overallocate their attention to distractors, as suggested by their breakdown in performance accuracy with increasing distractor salience. (3) Consequently, in subjects with ADHD and in the controls but not in alcohol-dependent subjects, performance accuracy was negatively associated with ADHD inattentive symptoms, suggesting that inattention seems to contribute to the emotional interference control deficit in ADHD but not in alcohol dependence.

With regard to task validity, all subjects displayed decreased performance accuracy in the 2-back condition compared with the 1-back condition as well as gradually decreasing performance accuracy with increasing distractor salience in the emotional version of the task. Thus, although the manipulation of working memory load and distractor salience was successful in the emotional version of the task, the effect of WM load was not found in the classical version. Despite sensitivity to WM deficits in the clinical groups, the less complex task may have been too easy to provoke significant differences between the conditions, and the effect of WM load may have become more apparent in the more complex task that included additional visual stimulation.

Longitudinal studies are needed to examine the stability of the emotional interference deficit in subjects with ADHD and in alcohol-dependent subjects. Because DLPFC integrity seems permanently impaired in subjects with ADHD [40], [41] but seems to vary more strongly as a function of abstinence duration in alcohol-dependent subjects [40], longitudinal studies would allow for the examination of recovery effects, which should be more prominent in the alcoholic group. Furthermore, longitudinal studies would allow for the examination of emotional interference control deficits resulting from prolonged alcoholism when working memory deficits become more severe.

In our study, we included males exclusively, but it would be interesting to include females in future studies. To the best of our knowledge, no research has addressed gender differences in emotional interference control in subjects with ADHD and in alcoholics, but a recently conducted study in healthy subjects using an emotional variant of the Stroop Task found no gender differences at the electrophysiological level [69]. However, there is some evidence for gender differences in the classical variant of the Stroop Task, which assesses cognitive interference control. This evidence demonstrates that females outperform males both in healthy subjects [70] and in subjects with ADHD [71]. However, the results still seem inconclusive for subjects with ADHD because other studies have found no gender differences [72], [73]. Functional imaging studies on emotion processing in healthy subjects suggest that females respond more strongly to negative emotional stimuli [74], [75], but males more strongly recruit regulatory areas [76]. The latter finding is also evident in self-ratings of emotional control, in which males report suppressing their emotions to a larger degree than do women [77]. Furthermore, it has been found that differences in brain structure between females and males are associated with differences in ER and emotion expression [78]. More precisely, females were found to have larger right orbitofrontal and ventromedial prefrontal cortex volumes, and a larger ventromedial prefrontal cortex volume is associated with using emotion suppression strategies less often and with a more intense emotion expression. Thus, future studies are required to analyze whether the stronger emotional responsiveness and less frequent use of emotion regulation strategies in females might facilitate an increased susceptibility to emotional interferences or whether their possibly superior interference control might compensate for this.

Beyond emotional interference control, future research should address the impact of gender differences on the development of comorbid affective disorders in subjects with ADHD and in alcoholics. For example, Biederman et al. [79], [80] found that girls with ADHD develop higher rates of major depressive disorder and anxiety disorders in their young adult years than do boys with ADHD, and Retz-Junginger, Rösler, Müller, and Retz [81] found that adult females with ADHD, who displayed elevated levels of emotional dysregulation when compared with males, were overrepresented among the clients of their outpatient services. In this sense, females with ADHD seem to be at particular risk for the development of affective disorders when compared with males with ADHD, which may be due to gender differences in emotion processing.

### Limitations

Some methodological limitations of our study need to be considered. First, we included only male subjects. Thus, our results cannot be generalized to female subjects. Second, although the exclusion of comorbid psychiatric disorders that may have affected test performance resulted in a higher specificity of our findings, the impact of these comorbidities on emotional interference control will have to be examined in future studies. The importance of both gender and comorbidity aspects is suggested, for example, by recent findings demonstrating that females with ADHD, when compared with males, display higher rates of comorbid mood disorders [47] and borderline personality disorder [46]; both of these disorders have emotional interference control deficits [82]–[88]. These findings suggest larger impairment in coping with emotional interferences in females than in males with ADHD. However, males and females with ADHD have been found to display differential brain activation patterns while performing the n-back task, with males, but not females, being impaired relative to controls [89]. These findings suggest that females with ADHD might be better able to block out emotional distractors [90], which might parallel the impact of higher relevant comorbidity. Third, it should be noted that in the present study, the probability to detect group differences was reduced due to limited statistical power. This limitation might be especially true for the classical n-back task and the surprise recognition memory task, for which trend effects have been found.

## Conclusions

The results of the present study suggest that subjects with ADHD and alcohol-dependent subjects share dysfunctions in a frontal-parietal-cerebellar network subserving working memory [91], [92], as measured by their impaired n-back task performance relative to controls. Additionally, we were able to demonstrate that both clinical groups display an emotional interference control deficit, and this deficit is even more pronounced in subjects with ADHD. Whereas the performance of subjects with ADHD deteriorated in the presence of low emotionally salient distractors, the performance of alcohol-dependent subjects did not deteriorate until highly salient distractors were presented. Based on our data, we speculate that in subjects with ADHD, differences in attention allocation processes (as indicated by higher distractor recognition rates and a lack of negative correlation between task performance and subsequent distractor identification) and a more general attention deficit (as indicated by a positive correlation between n-back task performance and clinical ratings of ADHD inattentive symptoms) contribute to the more pronounced deficit pattern. However, this hypothesis should be tested in future studies.

Considering the high comorbidity between ADHD and alcohol dependence, it remains unclear whether the emotional interference control deficit is more pronounced in subjects with both conditions. This is a question that should be addressed in future studies. Moreover, further research should consider how deficient emotional interference control is associated with behavioral markers of emotional dysregulation and adult comorbid psychopathology. In the case of emotion regulation, for example, it has been shown that children with more severe emotion dysregulation differ from those with milder impairment in terms of higher rates of unipolar and bipolar mood disorders, oppositional defiant disorders, and conduct disorders [93]. Because emotion regulation is preceded by the modulation of interfering emotionally laden stimuli in emotion processing and because emotional interference control seems to be deficient especially in subjects with ADHD, the regulatory deficit might mediate the link between impaired emotional interference control and adult psychopathology in subjects with the disorder.

## References

[1] American Psychiatric Association (2000) Diagnostic and statistical manual of mental disorders (4th ed., text rev.). Washington, DC: Author.

[2] Faraone SV, Biederman J, Mick E (2006) The age-dependent decline of attention deficit hyperactivity disorder: a meta-analysis of follow-up studies. Psychol Med 36: 159–165. 16420712 10.1017/S003329170500471X

[3] Faraone SV, Biederman J (2005) What is the prevalence of adult ADHD? Results of a population screening of 966 adults. J Attent Disord 2: 384–391. 10.1177/1087054705281478 16371661

[4] Kessler RC, Adler L, Barkley R, Biederman J, Conners CK, et al (2006) The prevalence and correlates of adult ADHD in the United States: results from the National Comorbidity Survey Replication. Am J Psychiatry 163: 716–723. 16585449 10.1176/appi.ajp.163.4.716 PMC2859678

[5] Biederman J, Mick E, Faraone SV (2000) Age-dependent decline of symptoms of attention deficit hyperactivity disorder: impact of remission definition and symptom type. Am J Psychiatry 157: 816–818. 10784477 10.1176/appi.ajp.157.5.816

[6] Biederman J, Petty CR, Woodworth KY, Lomedico A, Hyder LL, et al (2012) Adult outcome of attention-deficit/hyperactivity disorder: a controlled 16-year follow-up study. J Clin Psychiatry 73: 941–950. 22901345 10.4088/JCP.11m07529

[7] Rösler M, Casas M, Konofal E, Buitelaar J (2010) Attention deficit hyperactivity disorder in adults. World J Biol Psychiatry 11: 684–698. 20521876 10.3109/15622975.2010.483249

[8] Ohlmeier MD, Peters K, Te Wildt B, Zedler M, Ziegenbein M, et al (2008) Comorbidity of alcohol and substance dependence with attention-deficit/hyperactivity disorder (ADHD). Alcohol Alcohol 43: 300–304. 18326548 10.1093/alcalc/agn014

[9] Shekim WO, Asarnow RF, Hess E, Zaucha K, Wheeler N (1990) A clinical and demographic profile of a sample of adults with attention deficit hyperactivity disorder, residual state. Compr Psychiat 31: 416–425. 2225800 10.1016/0010-440x(90)90026-o

[10] Smith BH, Molina BSG, Pelham WE (2002) The clinically meaningful link between alcohol use and attention deficit hyperactivity disorder. Alcohol Res Health 26: 122–129.

[11] Lee SS, Humphreys KL, Flory K, Liu R, Glass K (2011) Prospective association of childhood attention-deficit/hyperactivity disorder (ADHD) and substance use and abuse/dependence: a meta-analytic review. Clin Psychol Rev 31: 328–341. 21382538 10.1016/j.cpr.2011.01.006 PMC3180912

[12] Wilens TE (2004) Impact of ADHD and its treatment on substance abuse in adults. J Clin Psychiatry 65: 38–45. 15046534

[13] Anastopoulos AD, Smith TF, Garrett ME, Morrissey-Kane E, Schatz NK, et al (2011) Self-regulation of emotion, functional impairment, and comorbidity among children with ADHD. J Atten Disord 15: 583–592. 20686097 10.1177/1087054710370567 PMC3355528

[14] Maedgen JW, Carlson CL (2000) Social functioning and emotional regulation in the attention deficit hyperactivity disorder subtypes. J Clin Child Psychol 29: 30–42. 10693030 10.1207/S15374424jccp2901_4

[15] Melnick SM, Hinshaw SP (2000) Emotion regulation and parenting in AD/HD and comparison boys: linkages with social behaviors and peer preference. J Abnorm Child Psychol 28: 73–86. 10772351 10.1023/a:1005174102794

[16] Sjöwall D, Roth L, Lindqvist S, Thorell LB (2013) Multiple deficits in ADHD: executive dysfunction, delay aversion, reaction time variability, and emotional deficits. J Child Psychol Psychiatry 54: 619–627. 23061803 10.1111/jcpp.12006 PMC3758957

[17] Walcott CM, Landau S (2004) The relation between disinhibition and emotion regulation in boys with attention deficit hyperactivity disorder. J Clin Child Adolesc Psychol 33: 772–782. 15498744 10.1207/s15374424jccp3304_12

[18] Retz W, Stieglitz RD, Corbisiero S, Retz-Junginger P, Rösler M (2012) Emotional dysregulation in adult ADHD: What is the empirical evidence? Expert Rev Neurother 12: 1241–1251. 23082740 10.1586/ern.12.109

[19] Fox HC, Hong KA, Sinha R (2008) Difficulties in emotion regulation and impulse control in recently abstinent alcoholics compared with social drinkers. Addic Behav 33: 388–394. 10.1016/j.addbeh.2007.10.002 18023295

[20] Thorberg FA, Lyvers M (2006) Negative Mood Regulation (NMR) expectancies, mood, and affect intensity among clients in substance disorder treatment facilities. Addict Behav 31: 811–820. 15993006 10.1016/j.addbeh.2005.06.008

[21] Fairholme CP, Nosen EL, Nillni YI, Schumacher JA, Tull MT, et al (2013) Sleep disturbance and emotion dysregulation as transdiagnostic processes in a comorbid sample. Behav Res Ther 51: 540–546. 23831496 10.1016/j.brat.2013.05.014 PMC3774794

[22] Kuvaas NJ, Dvorak RD, Pearson MR, Lamis DA, Sargent EM (2013) Self-regulation and alcohol use involvement: a latent class analysis. Addict Behav 39: 146–152. 24126205 10.1016/j.addbeh.2013.09.020 PMC4625554

[23] Sjoerds Z, van den Brink W, Beekman AT, Penninx BW, Veltman DJ (2013) Response inhibition in alcohol-dependent patients and patients with depression/anxiety: a functional magnetic resonance imaging study. Psychol Med 9: 1–13. 10.1017/S0033291713002274 24016382

[24] Barkley RA (2010) Deficient emotional self-regulation: a core component of attention-deficit/hyperactivity disorder. Journal of ADHD & Related Disorders, 1: , 5–37.

[25] Funahashi S (2001) Neuronal mechanisms of executive control by the prefrontal cortex. Neurosci Res 39: 147–165. 11223461 10.1016/s0168-0102(00)00224-8

[26] Hofmann W, Schmeichel BJ, Baddeley AD (2012) Executive functions and self-regulation. Trend Cogn Sci 16: 174–180. 10.1016/j.tics.2012.01.006 22336729

[27] Nigg JT, Casey BJ (2005) An integrative theory of attention-deficit/hyperactivity disorder based on the cognitive and affective neurosciences. Development and Psychopathology, 17: , 785–806. 10.1017/S0954579405050376 16262992

[28] Ochsner KN, Silvers JA, Buhle JT (2012) Functional imaging studies of emotion regulation: a synthetic review and evolving model of the cognitive control of emotion. Ann NY Acad Sci 1251: E1–E24. 23025352 10.1111/j.1749-6632.2012.06751.x PMC4133790

[29] Casey BJ, Durston S, Fossella JA (2001) Evidence for a mechanistic model of cognitive control. Clin Neurosci Res 1: 267–282.

[30] Miller EK, Cohen JD (2002) An integrative theory of prefrontal cortex activation. Ann Rev Neurosci 24: 167–202. 10.1146/annurev.neuro.24.1.167 11283309

[31] Norman DA, Shallice T (1986) Attention to action: Willed and automatic control of behavior. In R. JDavidson, C. ESchwartz & DShapiro (Eds.), Consciousness and self-regulation: Advances in research and theory (Vol. 4 , pp. 1–18). New York: Plenum Press.

[32] Abe M, Hanakawa T (2009) Functional coupling underlying motor and cognitive functions of the dorsal premotor cortex. Behav Brain Res 198: 13–23. 19061921 10.1016/j.bbr.2008.10.046

[33] Casey BJ, Thomas KM, Welsh TF, Livnat R, Eccard CH (2000). Cognitive and behavioral probes of developmental landmarks for use in functional neuroimaging. In MErnst & J. MRumsey (Eds.), Functional Neuroimaging in Child Psychiatry (pp. 155–168). New York: Cambridge University Press.

[34] Iordan AD, Dolcos S, Dolcos F (2013) Neural signatures of the response to emotional distraction: a review of evidence from brain imaging investigations. Front Hum Neurosci 7: 200. 23761741 10.3389/fnhum.2013.00200 PMC3672684

[35] Cromheeke S, Mueller SC (2013) Probing emotional influences on cognitive control: an ALE meta-analysis of cognition emotion interactions. Brain Struct Funct (accepted). 10.1007/s00429-013-0549-z 23563751

[36] Rottschy C, Langner R, Dogan I, Reetz K, Laird AR, et al (2012) Modelling neural correlates of working memory: a coordinate-based meta-analysis. Neuroimage 60: 830–846. 22178808 10.1016/j.neuroimage.2011.11.050 PMC3288533

[37] Marx I, Domes G, Havenstein C, Berger C, Schulze L, et al (2011) Enhanced emotional interference on working memory performance in adults with ADHD. World J Biol Psychiatry 12 (S1): 70–75. 10.3109/15622975.2011.599213 21905999

[38] Boonstra AM, Oosterlaan J, Sergeant JA, Buitelaar JK (2005) Executive functioning in adult ADHD: a meta-analytic review. Psychol Med 35: 1097–1108. 16116936 10.1017/s003329170500499x

[39] Schoechlin C, Engel RR (2005) Neuropsychological performance in adult attention-deficit hyperactivity disorder: meta-analysis of empirical data. Arch Clin Neuropsych 20: 727–744. 10.1016/j.acn.2005.04.005 15953706

[40] Stavro K, Pelletier J, Potvin S (2012) Widespread and sustained cognitive deficits in alcoholism: a meta-analysis. Addict Biol 18: 203–213. 22264351 10.1111/j.1369-1600.2011.00418.x

[41] Halperin JM, Trampush JW, Miller CJ, Marks DJ, Newcorn JH (2008) Neuropsychological outcome in adolescents/young adults with childhood ADHD: profiles of persisters, remitters and controls. J Child Psychol Psychiatry 49: 958–966. 18573145 10.1111/j.1469-7610.2008.01926.x PMC2646044

[42] Simon V, Czobor P, Bálint S, Mészáros A, Bitter I (2009) Prevalence and correlates of adult attention-deficit hyperactivity disorder: meta-analysis. Br J Psychiatry 194: 204–211. 19252145 10.1192/bjp.bp.107.048827

[43] Compton WM, Thomas YF, Stinson FS, Grant BF (2007) Prevalence, correlates, disability, and comorbidity of DSM-IV drug abuse and dependence in the United States: results from the national epidemiologic survey on alcohol and related conditions. Arch Gen Psychiatry 64: 566–576. 17485608 10.1001/archpsyc.64.5.566

[44] Goldstein RB, Dawson DA, Chou SP, Grant BF (2012) Sex differences in prevalence and comorbidity of alcohol and drug use disorders: results from wave 2 of the National Epidemiologic Survey on Alcohol and Related Conditions. J Stud Alcohol Drugs, 73: , 938–950. 10.15288/jsad.2012.73.938 PMC3469048 23036212

[45] Haberstick BC, Young SE, Zeiger JS, Lessem JM, Hewitt JK, et al (2013) Prevalence and correlates of alcohol and cannabis use disorders in the United States: results form the national longitudinal study of adolescent health. Drug Alcohol Depend 136: 158–161. 24440049 10.1016/j.drugalcdep.2013.11.022 PMC3963405

[46] Cumyn L, French L, Hechtman L (2009) Comorbidity in adults with attention-deficit hyperactivity disorder. Can J Psychiatry 54: 673–683. 19835674 10.1177/070674370905401004

[47] Groß-Lesch S, Dempfle A, Reichert S, Jans T, Geissler J, et al. (2013) Sex- and subtype-related differences in the comorbidity of adult ADHD. J Atten Disord 10.1177/1087054713510353 24196345

[48] Wittchen H, Zaudig M, Fydrich T (1997) Strukturiertes Klinisches Interview für DSM-IV (SKID). Göttingen: Hogrefe.

[49] Kuhl J, Kazén M (1997) Persönlichkeits- Stil- und Störungs-Inventar (PSSI): Handanweisung. Göttingen: Hogrefe.

[50] Barkley RA (1998) Attention-deficit hyperactivity disorder: a handbook for diagnosis and treatment, 2nd edn. New York: Guilford Press.

[51] Rösler M, Retz-Junginger P, Retz W, Stieglitz R-D (2008) HASE –Homburger ADHS-Skalen für Erwachsene. Göttingen: Hogrefe.

[52] Retz-Juninger P, Retz W, Blocher D, Weijers HG, Trott GE, et al (2002) Wender Utah rating scale. The short-version for the assessment of the attention-deficit hyperactivity disorder in adults. Nervenarzt 73: 830–838. 12215873 10.1007/s00115-001-1215-x

[53] Conners C, Erhardt D, Sparrow E (1998) The Conners Adult ADHD Rating Scale-Long Version (CAARS-S:L). Toronto: Multi-Health Systems.

[54] Barratt ES (1985) Impulsiveness subtraits: Arousal and information processing. In: Spence JT, Izard CE, editors.Motivation, emotion and personality. Amsterdam: North Holland/Elsevier Science.

[55] Retz-Junginger P, Retz W, Blocher D, Stieglitz RD, Georg T, et al (2003) Reliability and validity of the Wender-Utah-Rating-Scale short form. Retrospective assessment of symptoms for attention deficit/hyperactivity disorder. Nervenarzt 74: 987–993. 14598035 10.1007/s00115-002-1447-4

[56] Wetterling T, Kanitz R-D, Besters B, Fischer D, Zerfass B, et al (1997) A new rating scale for the assessment of the alcohol-withdrawal syndrome (AWS Scale). Alcohol Alcohol 32: 753–760. 9463730 10.1093/oxfordjournals.alcalc.a008326

[57] Tewes U (1994) Hamburg-Wechsler-Intelligenztest für Erwachsene – Revision 1991 (HAWIE-R). Bern: Huber.

[58] Lang PJ, Bradley MM, Cutberth BN (2008) International affective picture system (IAPS): Affective ratings of pictures and instruction manual. Technical Report A-8. Gainesville, FL: University of Florida.

[59] Rheinberg F, Vollmeyer R, Burns BD (2001) QCM: a questionnaire to assess current motivation in learning situations. Diagnostica 47: 57–66.

[60] Vollmeyer R, Rheinberg F (1998) Motivationale Einflüsse auf Erwerb und Anwendung von Wissen in einem computersimulierten System. Zeitschrift für Pädagogische Psychologie 12: 11–23.

[61] Murphy KR, Barkley RA, Bush T (2002) Young adults with attention deficit hyperactivity disorder: subtype differences in comorbidity, educational, and clinical history. J Nerv Ment Dis 190: 147–157. 11923649 10.1097/00005053-200203000-00003

[62] Taanila AM, Hurtig TM, Miettunen J, Ebeling HE, Mailanen IK (2009) Association between ADHD symptoms and adolescents' psychosocial wellbeing: a study of the Northern Finland birth cohort 1986. Int J Circumpolar Health 68: 133–142. 19517873 10.3402/ijch.v68i2.18324

[63] Carlson CL, Mann M, Alexander DK (2000) Effects of reward and response cost on the performance and motivation of children with AD/HD. Cognit Ther Res 24: 87–98.

[64] Niederhofer H (2008) Attributions for school success and failure by adolescent students with and without attention deficit hyperactivity disorder. Psychol Rep 102: 616–620. 18567234 10.2466/pr0.102.2.616-620

[65] Rucklidge J, Brown D, Crawford S, Kaplan B (2007) Attributional styles and psychosocial functioning of adults with ADHD. J Atten Disord 10: 288–298. 17242424 10.1177/1087054706289942

[66] Strand MT, Hawk LW, Bubnik M, Shiels K, Pelham WE (2012) Improving working memory in children with attention-deficit/hyperactivity disorder: the separate and combined effects of incentives and stimulant medication. J Abnorm Child Psychol 40: 1193–1207. 22477205 10.1007/s10802-012-9627-6 PMC3422611

[67] Cubillo A, Smith AB, Barrett N, Giampietro V, Brammer M, et al (2013) Drug-specific laterality effects on frontal lobe activation of atomoxetine and methylphenidate in attention deficit hyperactivity disorder boys during working memory. Psychol Med 44: 633–646. 23597077 10.1017/S0033291713000676

[68] Wong CG, Stevens MC (2012) The effects of stimulant medication on working memory functional connectivity in attention-deficit/hyperactivity disorder. Biol Psychiatry 71: 458–466. 22209640 10.1016/j.biopsych.2011.11.011 PMC4120250

[69] Putman P, Arias-Garcia E, Pantazi I, van Schie C (2012) Emotional Stroop interference for threatening words is related to reduced EEG delta-beta coupling and low attentional control. Int J Psychophysiol 84: 194–200. 22353580 10.1016/j.ijpsycho.2012.02.006

[70] Van der Elst W, Van Boxtel MP, Van Breukelen GJ, Jolles J (2006) The Stroop color-word test: influence of age, sex, and education; and normative data for a large sample across the adult age range. Assessment 13: 62–79. 16443719 10.1177/1073191105283427

[71] Bálint S, Czobor P, Comlósi S, Mészáros Á, Simon V, et al (2009) Attention deficit hyperactivity disorder (ADHD): gender- and age-related differences in neurocognition. Psychol Med 39: 1337–1345. 18713489 10.1017/S0033291708004236

[72] Rucklidge JJ, Tannock R (2002) Neuropsychological profiles of adolescents with ADHD: effects of reading difficulties and gender. J Child Psychol Psychiatry 43: 988–1003. 12455921 10.1111/1469-7610.00227

[73] Van Mourik R, Oosterlaan J, Sergeant JA (2005) The Stroop revisited: a meta-analysis of interference control in AD/HD. J Child Psychol Psychiatry 46: 150–165. 15679524 10.1111/j.1469-7610.2004.00345.x

[74] Domes G, Schulze L, Böttger M, Grossmann A, Hauenstein K, et al (2010) The neural correlates of sex differences in emotional reactivity and emotion regulation. Hum Brain Mapp 31: 758–769. 19957268 10.1002/hbm.20903 PMC6871188

[75] Stevens JS, Hamann S (2012) Sex differences in brain activation to emotional stimuli: a meta-analysis of neuroimaging studies. Neuropsychologia 50: 1578–1593. 22450197 10.1016/j.neuropsychologia.2012.03.011

[76] Mak AK, Hu ZG, Zhang JX, Xiao Z, Lee TM (2009) Sex-related differences in neural activity during emotion regulation. Neuropsychologia 47: 2900–2908. 19555702 10.1016/j.neuropsychologia.2009.06.017

[77] Melka SE, Lancaster SL, Bryant AR, Rodriguez BF (2011) Confirmatory factor and measurement invariance analyses of the emotion regulation questionnaire. J Clin Psychol 67: 1283–1293. 21928369 10.1002/jclp.20836

[78] Welborn BL, Papademetris X, Reis DL, Rajeevan N, Bloise SM, et al (2009) Variation in orbitofrontal cortex volume: relation to sex, emotion regulation and affect. Soc Cogn Affect Neurosci 4: 328–339. 20019072 10.1093/scan/nsp028 PMC2799952

[79] Biederman J, Monuteaux M, Mick E, Spencer T, Wilens T, et al (2006) Young adult outcome of attention deficit hyperactivity disorder: a controlled 10-year follow-up study. Psychol Med 36: 167–179. 16420713 10.1017/S0033291705006410

[80] Biederman J, Petty CR, Monuteaux MC, Fried R, Byrne D (2010) http://www.ncbi.nlm.nih.gov/pubmed?term=Mirto%20T%5BAuthor%5D&cauthor=true&cauthor_uid=20080984 et al Adult psychiatric outcomes of girls with attention deficit hyperactivity disorder: 11-year follow-up in a longitudinal case-control study. Am J Psychiatry 167: 409–417. 20080984 10.1176/appi.ajp.2009.09050736

[81] Retz-Junginger P, Rösler M, Müller R, Retz W (2012) Impact of gender on the utilization of outpatient health service for adult ADHD. Psychiatr Prax 39: 345–348. 23044848 10.1055/s-0032-1305193

[82] Dichter GS, Felder JN, Smoski MJ (2009) Affective context interferes with cognitive control in unipolar depression: an fMRI investigation. J Affect Disord 114: 131–142. 18706701 10.1016/j.jad.2008.06.027 PMC2656256

[83] Domes G, Winter B, Schnell K, Vohs K, Fast K, et al (2006) The influence of emotions on inhibitory functioning in borderline personality disorder. Psychol Med 36: 1163–1172. 16700964 10.1017/S0033291706007756

[84] Joormann J, Nee DE, Berman MG, Jonides J, Gotlib IH (2010) Interference resolution in major depression. Cogn Affect Behav Neurosci 10: 21–33. 20233953 10.3758/CABN.10.1.21 PMC2845922

[85] Mensebach C, Wingenfeld K, Driessen M, Rullkoetter N, Schlosser N, et al (2009) Emotion-induced memory dysfunction in borderline personality disorder. Cogn Neuropsychiatry 14: 524–541. 19894145 10.1080/13546800903049853

[86] Rey G, Desseilles M, Favre S, Dayer A, Piguet C, et al (2014) Modulation of brain response to emotional conflict as a function of current mood in bipolar disorder: preliminary findings from a follow-up state-based fMRI study. Psychiatr Res 223: 84–93. 10.1016/j.pscychresns.2014.04.016 24862389

[87] Segrave RA, Thomson RH, Cooper NR, Croft RJ, Sheppard DM, et al (2012) Emotive interference during cognitive processing in major depression: an investigation of lower alpha 1 activity. J Affect Disord 141: 185–193. 22534463 10.1016/j.jad.2012.03.004

[88] Wang KS, Labar M, Smoski MZ, Rosenthal F, Dolcos TR, et al (2008) Prefrontal mechanisms for executive control over emotional distraction are altered in major depression. Psychol Res 163: 143–155. 10.1016/j.pscychresns.2007.10.004 PMC2553159 18455373

[89] Valera EM, Brown A, Biederman J, Faraone SV, Makris N, et al (2010) Sex differences in the functional neuroanatomy of working memory in adults with ADHD. Am J Psychiatry 167: 86–94. 19884224 10.1176/appi.ajp.2009.09020249 PMC3777217

[90] Burgess GC, Depue BE, Ruzic L, Willcutt EG, Du YP, et al (2010) Attentional control activation relates to working memory in attention-deficit/hyperactivity disorder. Biol Psychiatry 67: 632–640. 20060961 10.1016/j.biopsych.2009.10.036 PMC2953472

[91] Hautzel H, Mottaghy FM, Specht K, Müller HW, Krause BJ (2009) Evidence of a modality-dependent role of the cerebellum in working memory? An fMRI study comparing verbal and abstract n-back tasks. Neuroimage 47: 2073–2082. 19524048 10.1016/j.neuroimage.2009.06.005

[92] Schmidt H, Jogia J, Fast K, Christodoulou T, Haldane M, et al (2009) No gender differences in brain activation during the N-back task: An fMRI study in healthy individuals. Hum Brain Mapp 11: 3609–3615. 10.1002/hbm.20783 PMC6870785 19387979

[93] Biederman J, Petty CR, Day H, Goldin RL, Spencer T, et al (2012) Severity of the aggression/anxiety-depression/attention child behavior checklist profile discriminates between different levels of deficits in emotional regulation in youth with attention-deficit hyperactivity disorder. J Dev Behav Pediatr 33: 236–243. 22278125 10.1097/DBP.0b013e3182475267 PMC3319866

